# A Comprehensive Evaluation of Mobility: Validation of the Functional Ambulation and Stair Test in Older Adults

**DOI:** 10.3390/jcm15124782

**Published:** 2026-06-19

**Authors:** Anson B. Rosenfeldt, Elizabeth Claire Weyman Heller, Eric Zimmerman, Sara Davidson, John Gardner, Grant Alberts, Benjamin Broz, Jordan Klein, Louie Sutte, Emily Hopkins, Jay L. Alberts

**Affiliations:** 1 Department of Biomedical Engineering, Cleveland Clinic Research, Cleveland Clinic, 9500 Euclid Ave., Cleveland, OH 44195, USA; 2 Department of Chemical and Biomedical Engineering, Cleveland State University, 2121 Euclid Ave., Cleveland, OH 44115, USA; 3 Center for Neurological Restoration, Neurological Institute, Cleveland Clinic, 9500 Euclid Ave., Cleveland, OH 44195, USA

**Keywords:** older adults, stairs, gait, turning, dual-task, validation

## Abstract

**Background/Objectives:** Falls have devastating consequences for older adults. The Functional Ambulation and Stair Test (FAST) was developed to characterize older adult mobility and eventual fall risk. This project aimed to determine the criterion validity of the FAST assessment by comparing the relationship between FAST outcomes and existing gold-standard clinical assessments of mobility and fall risk. A secondary aim was assessing the FAST’s capacity to elicit dual-task effects in older adults. **Methods:** The FAST is a multi-faceted mobility assessment combining stair navigation, turning and level-ground walking; total time and time spent in each phase are the calculated outcomes. Data from 199 older adults completing the FAST, Berg Balance Scale (BBS), Timed Up and Go (TUG), and Ten Meter Walk Test (10MWT) at comfortable and fast speed were evaluated. Relationships between the FAST and clinical outcomes were evaluated with Spearman’s correlations. The FAST and TUG were assessed under single- and dual-task conditions; linear mixed models evaluated the dual-task effects for overall FAST time and each phase. **Results:** Spearman’s correlations between the FAST and the BBS, TUG, 10MWT comfortable and 10MWT fast were −0.65, 0.88, −0.79, and −0.83, respectively. Participants experienced an 8.6% and 13.2% dual-task cost in the FAST and TUG, respectively. The greatest dual-task cost during the FAST was in the gait initiation, walking, and wide turn phases. **Conclusions:** Agreement between the FAST and gold-standard clinical mobility assessments confirms the criterion validity of the FAST. Delineation of mobility phases via the FAST offers insight into specific mobility deficits. Future work is ongoing to evaluate the FAST as a fall risk assessment in older adults.

## 1. Introduction

Falls are a common cause of traumatic brain injury [1], fracture [2], and are the leading cause of unintentional death for older adults [3,4,5,6]. Acutely, falls can result in injury, functional decline, loss of independence, and, in some cases, death [7]. Long-term sequelae of falls include fear of falling and activity avoidance [8], decreased quality of life [9], institutionalization [10], and come with accompanying steep financial consequences. [11,12]. Preventing falls is a top priority in the U.S. and globally [13,14]. Ideally, individuals at risk of falls would be prospectively identified, and appropriate proactive, impairment-specific interventions would be initiated. At present, the best predictor of a future fall is a previous one [15], thus, the health care community is missing a critical interventional period to prevent a potentially catastrophic or impactful first fall. A gap in evaluating older adult mobility is the lack of a rapid assessment that objectively quantifies specific mobility challenges that are known to trigger mobility impairment and falling in older adults.

Three frequent scenarios associated with falling include flat surface walking [16], stair navigation [17], and turning [16]. Walking is the most common activity associated with falls in older adults, with over 50% of falls occurring during ambulation [17]. Comprising approximately 15% of total falls in older adults [17], stair-related injuries result in over 1 million emergency room visits annually in the United States [18]. Stair-related injuries are on the rise; a 2018 study reported that stair-related injuries across all age groups increased by 24% from 1996 to 2012 [18], with older adults experiencing the highest injury rate. Between 2012 and 2021, stair-related upper extremity fractures in older adults increased by 56% and hospitalizations associated with stair-related injuries increased by 38% [19]. Older adults are particularly susceptible to stair-related falls due to age-related declines in cardiovascular health, musculoskeletal function, and vision including impaired depth perception, resulting in slow, less stable stair ascent strategies with poor foot clearance [20].

Turning is a complex motor task that carries a high risk of falling in older adults [16]. Turning comprises up to 50% of the proportion of steps taken during activities of daily living performance [21]; an observational study in a free-living environment found that older adults perform, on average, 866 turns per day [22]. Falling during a turn can be detrimental and is eight times more likely to result in a hip fracture than falls during straight line walking [23]. Age-related changes in controlling one’s center of mass over a dynamic base of support contribute to increased turn time, increased number of steps during the turn, and movement hesitancy compared to young adults [24]. 

Fusing three high-risk fall activities—walking, stair navigation, and turning [16,17]—into a single, rapid physical evaluation that can be completed under single- and dual-task conditions may help to better understand fall risk and specific aspects of function contributing to that risk. The Functional Ambulation and Stair Test (FAST) was developed to objectively quantify walking, stair navigation and turning in a single assessment. The FAST combines equipment is available in therapy and medical offices with low-cost photocell technology that has been used in motor control research for decades [25]. This project aimed to validate the FAST as a measure of functional mobility by comparing FAST temporal outcomes to valid and reliable gold-standard mobility and fall risk assessments, the Berg Balance Scale (BBS), the Timed Up and Go (TUG) test, and walking speed. It was hypothesized that FAST outcomes would be related to the existing battery of mobility and fall risk assessments, as the FAST contains important elements of each test. Considering the age-related dual-task declines and their potential impact on increased fall rates [26], a secondary aim was to determine if the FAST was a suitable paradigm to elicit dual-task costs (DTC) in older adults.

## 2. Materials and Methods

### 2.1. Participants 

Between June and August 2025, community-dwelling older adults were recruited from surrounding communities and independent living facilities associated with a Continuing Care Retirement Community (CCRC) model in northeast Ohio. Notably, independent living facilities are facilities where residents have their own private living space (i.e., condominiums, apartments, or attached houses). The outdoor space is generally cared for by staff, but no assistance is provided for maintaining the indoor space or daily living tasks. Inclusion criteria were: (1) at least 62 years of age, (2) the ability to walk independently or with supervision, with or without a cane, (3) the ability to ascend and descend three steps, and (4) the ability to follow 2-step commands. Exclusion criteria were: (1) clinical diagnosis of dementia, (2) neurological disease that impacts motor/cognitive function, (3) musculoskeletal impairment that makes the participant unable to perform the assessments, and (4) uncorrected vision or hearing impairments that would make following instructions difficult. Participants were part of a longitudinal observational project related to understanding functional mobility and future fall risk including risk of transitioning from a non-faller to a recurrent faller. The data collection session analyzed in this manuscript was from a baseline evaluation of functional mobility, fall risk and general health status. This study was approved by the Cleveland Clinic Institutional Review Board, and all participants completed the informed consent process prior to participation. 

### 2.2. Study Protocol 

All data were gathered during a one-hour data collection session. During the session, participants completed a demographic questionnaire, Saint Louis University Mental Status Examination (SLUMS; questionnaire that consists of 11 questions that assesses orientation, memory, attention, and executive function; the maximum score is 30 points, with a higher score indicating better performance) [27], and performed four mobility assessments: the BBS, the TUG test, the Ten Meter Walk Test (10MWT), and the FAST. The order of mobility testing was alternated between participants (i.e., Participant 1 began with the BBS, Participant 2 with the TUG, etc.). To evaluate the secondary aim, the TUG and the FAST were performed under single- and dual-task conditions. 

### 2.3. Clinical Assessments 

The BBS is a valid and reliable test of balance and fall risk for adults [28,29]. The test consists of 14 items including static standing balance, retrieving an item from the floor, and turning. A clinician rates the performance of each task from 0 to 4 with a higher score indicating better performance (score ranges between 0 and 56 points).

The TUG is a valid and reliable test of functional mobility. To complete the TUG, a participant stands up from a chair, walks three meters at a comfortable pace, turns 180 degrees, walks back to the chair, and sits down [30,31,32]. A faster time indicates greater functional mobility. Two trials of the TUG were performed under single- and dual-task conditions. Consistent with the TUG-Cognitive assessment [33], for the dual-task condition the participant was asked to count backward by threes from a randomly selected number between 100 and 200. The average of the two trials for each condition was used in the analysis.

Gait speed (m/s) was assessed using the 10MWT. The participants walked on a 10 m course that included a 2 m acceleration, 6 m timed distance, and 2 m deceleration [34]. Two trials were performed at a comfortable pace, followed by two trials at a fast pace. The average of the two trials at each pace was used in the analysis.

**Figure 1:**
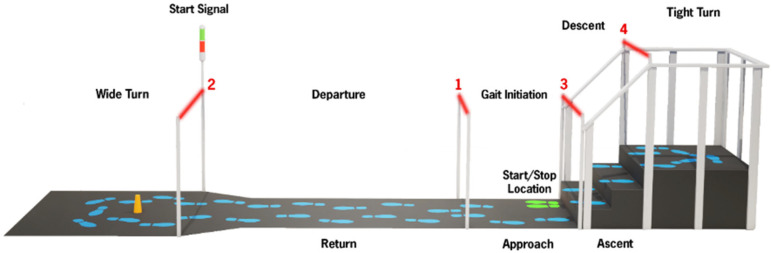
Illustration of the Functional Ambulation and Stair Test (FAST). The participant began at the base of the stairs (green footprints). Upon receiving the start signal denoted by the change of the light from red to green, the participant initiated gait (Gait Initiation Phase; start signal to sensor #1) walked away from the stairs (Departure Phase, 2.5 m; sensor #1 to #2), turned around a cone (Wide Turn Phase; sensor #2 initial to final), returned to the base of the stairs (Return Phase, 2.5 m; sensor #2 to #1), and approached the stairs (Approach Phase, 1 m; sensor #1 to #3), ascended the stairs (Ascent Phase; sensor #3 to #4), performed a turn at the top of the stairs (Tight Turn Phase; sensor #4 initial to final), and descended the stairs back to the starting position (Descent Phase; sensor #4 to #3). The blue footprints represent the movement path of the participant while performing the FAST; the start/stop location is represented by the green footprints. As the participant passed through the four photoelectric sensors (red), the time spent in each phase was automatically calculated.

The FAST is a multi-faceted mobility assessment that combines stair navigation, turning, and level-ground walking (Figure 1). A three-step wooden stair unit (Hausmann Industries, Northvale, NJ, USA), was used for the stair ambulation portion of the FAST. Briefly, three steps were selected as single-story homes may have between 2 and 3 steps connecting the garage to the main dwelling of the house. The 3-step staircase is a common piece of equipment in rehabilitation centers.

Four photoelectric sensor gates (Contrinex Inc., Coppel, TX, USA) provide temporal data for the eight phases of the FAST. As illustrated in Figure 1, the starting position is 1 m in front of the stairs. The trial begins when the start signal changes from “red” to “green”. The FAST consists of eight distinct phases: (1) Gait Initiation: interval between start signal turning green and movement initiation based on breaking sensor #1; (2) Departure: interval walking 2.5 m between sensor #1 and #2; (3) Wide Turn: interval between initial and final breaking of sensor #2 as a 180-degree turn is performed in a width of 1.5 m; (4) Return: interval walking 2.5 m between sensor #2 and #1; (5) Approach: 1 m interval approaching the stairs defined by the time between breaking sensors #1 and #3; (6) Ascent: interval to ascend three steps between sensor #3 and #4; (7) Tight Turn: interval to complete a 180-degree turn on the 0.9 m wide platform of the stairs as calculated by the initial and final breaking of photocell #4; and (8) Descent: interval to descend the three steps between breaking sensors #4 and #3. The FAST completion time was calculated as the duration between the trigger light turning green and breaking sensor #3 after descending the steps. The data collection and temporal calculation program was written in Python (version 3.11.2, Python Software Foundation, Beaverton, OR, USA) and the data were immediately displayed for the administrator on the tablet device and stored for future analysis. 

Participants were instructed to perform the task at a comfortable pace and were permitted to use the handrails and an assistive device (i.e., a cane) as they would in a real-world situation. Prior to the trial, an administrator demonstrated the task and the participant performed a familiarization trial to ensure understanding. Following a familiarization trial, participants completed six trials: three single-task and three dual-task trials. The order of the single- and dual-task were performed in alternating order to reduce learning effects. Identical to the TUG, the Serial 3s task was used as the secondary cognitive task during the dual-task trials. Prior to initiating the dual-task trials, participants verbalized 3–5 responses to the Serial 3s task while standing to ensure understanding of the task. During the dual-task scenario, no task-specific prioritization instructions were provided. To ensure the participant was engaging with the cognitive task, a dual-task trial was repeated if a participant failed to respond to the Serial 3s task. The accuracy of Serial 3s was not recorded. The overall time difference between the three FAST trials was 400 ms and variability of trial times was consistent within each subject (ICC_3,1_ = 0.97 for single-task total times); thus, data were collapsed across trials for each participant.

### 2.4. Statistical Analysis 

To address the primary aim of FAST validation with established gold-standard outcome metrics, Spearman’s correlations were used to determine the relationship between the single-task FAST and other gold-standard outcome metrics (BBS, single-task TUG, and 10MWT) to allow for nonlinear monotonic relationships and limit the influence of extreme values. Confidence intervals were calculated for the correlations using the bootstrap methodology, in which data were resampled 2000 times, the correlation was calculated on each resample, and then a 95% confidence interval was calculated from the distribution of resampled correlations. 

To address the secondary aim, linear mixed models (LMM) were used to assess the presence and estimate the magnitude of a median DTC in FAST completion time and for each phase. The DTC was defined as the percent difference in dual-task conditions as compared to single-task conditions [35]. In the assessment of an overall DTC in the FAST, completion time was modeled using a fixed effect of task and random intercepts by participant. For comparison, TUG DTC was modeled in the same manner. The DTC in FAST and TUG completion time were further quantified with Hedges’ g effect size, calculated on the log-scale using the pooled standard deviation. In the assessment of DTC by FAST phase, individual phase times were modeled with fixed effects of task, phase, and the interaction of task and phase, and random intercepts of participant. The comparison of task within each phase was adjusted for multiple comparisons using the Holm–Bonferroni method across phase comparisons. Due to the skewness of trial data, times were log-transformed in the LMM analysis. The LMM estimates were back-transformed from the log-scale and, as FAST and TUG times were approximately log-normally distributed, represent medians and relative differences. Demographic and performance data were otherwise summarized with means and standard deviations (SD) if continuous and approximately normally distributed, median and quartiles if continuous and skewed, and with frequency counts and percentages if categorical. The analyses were conducted using RStudio 2026.01.0, R version 4.5.2. 

## 3. Results

A total of 202 individuals were enrolled; three were unable to complete the entire test battery due to cognitive or physical impairments. Demographics for the remaining 199 participants are provided in Table 1.

### 3.1. Validation of FAST with Clinical Outcomes 

**Figure 2:**
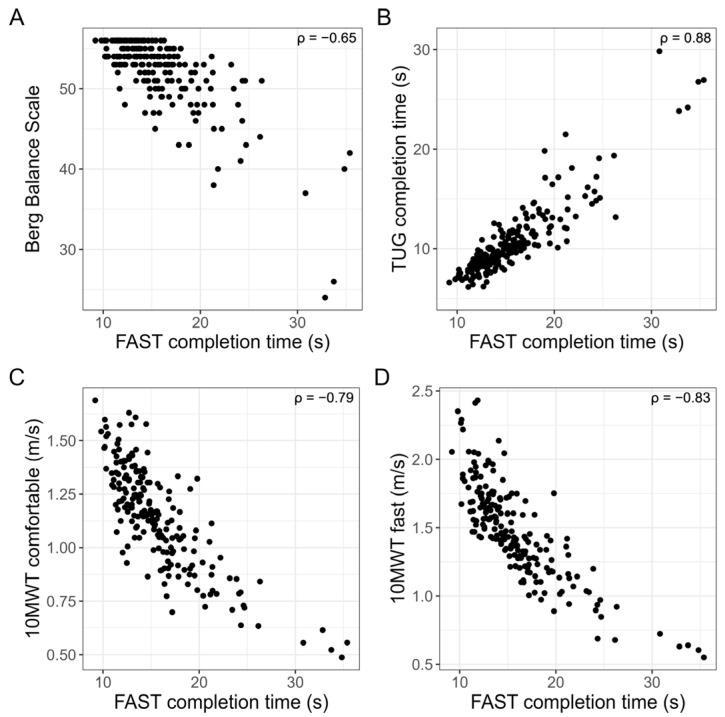
Spearman’s correlations between the single-task FAST and each of the BBS, single-task TUG, 10MWT comfortable and 10MWT fast were moderate to very strong at −0.65 (**A**), 0.88 (**B**), −0.79 (**C**) and −0.83 (**D**), respectively.

Performance outcomes for the clinical tests are provided in Table 2. As shown in Figure 2, FAST completion time was related to outcomes from gold-standard clinical tests. Spearman’s correlation between the single-task FAST completion time and BBS was −0.65 (95% CI: −0.73, −0.56, *p* < 0.001) (Figure 2A). Single-task FAST completion time had a correlation of 0.88 (0.84, 0.91) with single-task TUG (*p* < 0.001) (Figure 2B). Similarly, FAST completion time was highly correlated with the 10MWT under comfortable and fast-paced conditions, −0.79 (−0.85, −0.72) and −0.83 (−0.88, −0.76), respectively (*p* < 0.001) (Figure 2C,D). 

### 3.2. Validation of FAST to Induce a Dual-Task Cost 

The FAST completion time (median (95% CI)) significantly slowed (*p* < 0.001) from 15.2 (14.7, 15.8) to 16.5 (15.9, 17.2) seconds when moving from single- to dual-task conditions with a DTC of 8.6% (7.4%, 9.9%, Hedges’ g = −0.30).

The TUG completion time also significantly slowed (*p* < 0.001) from 10.3 (9.9, 10.8) to 11.7 (11.2, 12.2) seconds during single- and dual-task conditions, respectively, with a DTC of 13.2% (11.4%, 15.0%, Hedges’ g = −0.39). The correlation between the DTC on the FAST and the TUG was 0.50 (0.39, 0.61, *p* < 0.001). 

**Figure 3:**
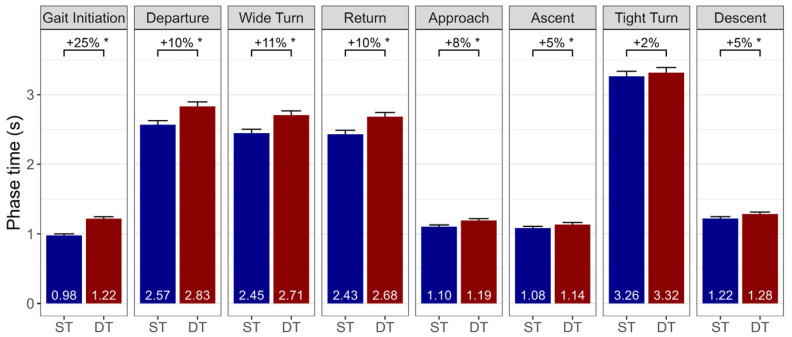
Single-task (ST; blue) and dual-task (DT; red) performance time during each phase of the FAST presented as median (SE). The median values (seconds) for each phase are provided at the bottom of each bar. The dual-task cost (DTC) percentage is provided for each phase above the bars; a higher number indicated a greater DTC (i.e., a slower performance under dual-task conditions). * denotes a significant (*p* < 0.05) DTC within a given phase.

For the FAST phase times, dual-task times were overall significantly longer than single-task times (*p* < 0.001), and the magnitude of DTC changed by phase (*p* < 0.001). The DTC ranged from 2% to 25% by phase, with the greatest DTC occurring in the Gait Initiation Phase and the smallest DTC in the Tight Turn Phase (Figure 3). The Tight Turn Phase was the only phase not significantly prolonged from single- to dual-task conditions.

## 4. Discussion

The FAST provides valid assessment of complex mobility and elicits dual-task costs in community-dwelling older adults. Correlations between the FAST and the BBS, TUG, and 10MWT ranging from moderate (−0.65) to very strong (0.88) [36] support its criterion validity with existing clinical gold-standard fall risk and functional mobility assessments. While other standardized mobility fall assessment tools combine phases of functional mobility into a single score or a total time, the FAST is unique as it provides objective and quantitative temporal outcomes for the entire mobility task and each movement phase. Providing specific temporal data for walking, stair navigation (ascent and descent), and turning provides greater precision in a patient’s specific functional mobility challenges and may facilitate patient-specific interventions and address these declines.

Dual-tasking during the FAST resulted in an overall slowing of 8.6% compared to single-task conditions. Thus, the FAST paradigm when combined with the Serial 3s task does provoke a DTC. The deficit in dual-task performance is consistent with other studies reporting DTC in older adults during postural control tasks [37], gait and turning tasks [38], and obstacle navigation [39]. The importance of phase delineation was supported by the range of DTC (2–25%) across the phases, suggesting that each phase captures a unique aspect of motor control and function that are independently impacted by DTC.

The greatest DTC was in the Gait Initiation Phase (25% loss). Gait initiation may be a key component to fall risk assessment, as older adults with a history of falls exhibit greater anticipatory postural adjustments during gait initiation than those without falls [40]. Mounting evidence suggests that gait initiation tasks under dual-task conditions may further provoke postural instability and provide insight into fall risk [41]. Melzer and colleagues reported a ~100% increase in step initiation time in older adults under dual-task conditions compared to single-task [42]. In the Melzer study, the slowing was present throughout the entire initiation process (i.e., cognitive processing, weight shifting, and swing initiation) [42], suggesting that afferent sensorimotor processing, anticipatory postural adjustments, and motor execution are all impaired under dual-task conditions. Gait initiation is not measured, either objectively or subjectively, in clinical measures such as the BBS, TUG, and 10MWT. The FAST objectively quantifies this important motor phase, providing additional insight into postural control and potential fall risk. 

The Departure, Wide Turn, and Return Phases demonstrated a significant 10–11% DTC. These phases mirror the ambulatory phases of the TUG, which exhibited a similar 13% DTC. The Approach Phase, which encompassed the transition from level ground to the first stair, resulted in an 8% DTC. Separating the Approach Phase from the Ascent Phase provides value, as even young healthy adults exhibit different locomotor strategies and reaction time behavior during stair transitions (e.g., approaching the stairs) compared to stair navigation [43,44]. The DTC during stair navigation was smaller (5%), but it did reach statistical significance, while the Tight Turn Phase at the top of the stairs was negligible. While Zhang and colleagues reported a modest (~8%) DTC in older adults during stair descent speed [45], Salzman and colleagues reported no difference in stair navigation speed in older adults under single- and dual-task conditions, but did report a significant increase in prefrontal cerebral oxygenation under the dual-task stair condition [46]. The physiological response in the absence of temporal changes suggests that completion time alone may not capture the taxing nature of dual-task performance. Physiologic and refined biomechanical analyses may be necessary to fully realize DTC. Our previous work [47] and another study [48] support the use of kinetic data to differentiate between single- and dual-task conditions during stair navigation tasks. The addition of kinetic variables such as center of pressure may further discern fall vulnerabilities in an older adult population.

The strengths of this study include a large sample size and comprehensive suite of clinical mobility outcomes. It is fully appreciated that new approaches in assessment, especially those using some level of technology, must be conducive to integration into existing clinical workflows. The FAST was created due to limitations in existing models used to evaluate mobility and predict risk of falling. Our Develop with Clinical Intent Model [49] was utilized in the creation of the FAST protocol. The widespread integration of objective and quantitative measures of biomechanics and motor control has not occurred in typical rehabilitation practices. Often the cost or space requirements of a new test or technology prevent integration into busy clinical workflows. For example, traditional 3D motion capture systems often exceed $100K, require dedicated space and technicians, and require a prolonged time to process the data which prevents using it in the same patient visit. Wearable sensors generally face similar challenges in terms of integrating data into busy clinical workflows. Theoretical and position papers abound with proposed models of integrating wearable and other technology into patient assessment and rehabilitation planning [50,51]; however, uptake of rehabilitation technology is limited [52]. A fundamental challenge in these models is a lack of appreciating the number of elements that influence clinical workflows (e.g., administrative, human resources, financial, documentation, IT, and cybersecurity, to name a few). In its current form, the FAST system would require approximately $1200.00 capital investment (stairs, photoelectric sensors and tablet device). One could theoretically replicate the FAST set-up without the photoelectric sensors and use a stopwatch with a lap function to identify the temporal aspects of each phase and overall time to complete. Space requirements are moderate as the test requires a 4.5 m by 2 m space; notably, it is not required that this space be exclusively dedicated to the FAST paradigm. Using our successful clinical implementation models [49,53,54] as a guide, it is envisioned FAST could be integrated into a rehabilitation practice in large and small medical centers and private practice rehabilitation groups. Further, current efforts are underway to integrate the FAST protocol into the primary care provider space, which delivers care to geriatric patients as a rapid mobility screen. The advantage of the FAST approach is that it does not require a rehabilitation specialist to administer and the temporal outcome data are relatively straightforward to understand. The results from the ongoing, longitudinal fall tracking project will provide additional clinical context and potential fall risk models using FAST data that providers can use to stratify risk and make more patient specific recommendations to therapists treating these patients.

Performances on the BBS, TUG, and walking speed are consistent with established normative values for 60–90-year-olds [31]. The sample was recruited from diverse geographical regions throughout northeast Ohio, yet most of the sample identified as white. Integration into clinical workflows at the Cleveland Clinic and its regional hospitals provides an opportunity to address the lack of diversity. A variety of clinical assessments exist that assess gait, mobility, and balance in older adults. The BBS and the TUG are generally considered gold-standard mobility and fall risk assessments due to their well-established reliability [55,56,57], validity [56,58], and fall prediction cut-off scores [29,33,58]. Despite strong psychometric properties, the BBS is limited by a subjective, ordinal scoring scale, lack of gait assessment, and need for highly trained administrators (physical or occupational therapists). The TUG provides a single outcome (time to complete) and provides limited insight into specific mobility deficits as straight-line walking, postural transitions and turning are all combined. Numerous standardized mobility and fall assessments, including the functional gait assessment (FGA) [59] and dynamic gait index (DGI) [60], have been validated using the BBS, TUG, and gait speed with correlations in the 0.6–0.9 range—nearly identical to the correlations reported in this FAST validation report. Future directions may consider comparison with the functional gait assessment (FGA), one of the few clinical outcome metrics that includes a stair task [61]. Future directions for the FAST include test/retest reliability and fall prediction validity. For the latter, an on-going project is prospectively tracking falls over a 12-month period in the same group of older adults to determine the fall predictive validity of the FAST. Further, similar stair, turning, and gait paradigms are being assessed in individuals with Parkinson’s disease [47].

## 5. Conclusions

Moderate to strong correlations between the FAST and the BBS, TUG, and 10MWT suggest that the FAST is a valid measure of functional mobility. Further, the FAST was effective in provoking DTC in older adults. A unique aspect of the FAST is providing detailed temporal outcomes for each phase of movement. The results indicate that the Gait Initiation Phase was most susceptible to DTC, providing novel insight into motor control under dual-task conditions in this population. Providing specific temporal data for gait initiation, walking, transitional movements, stair ascent and descent, and turning has the potential to provide insight into mobility deficits and refine rehabilitation goals.

## Figures and Tables

**Table 1:** Participant demographics (N = 199).

Variable	Value
Age	77.5 (8.4)
Sex, Female	138 (69.3%)
Race	
Asian	3 (1.5%)
Black or African-American	7 (3.5%)
White	189 (95.0%)
Body Mass Index (BMI)	26.8 (5.37)
Education, years	16.3 (2.77)
Marital Status	
Divorced	22 (11.1%)
Domestic partnership	2 (1.0%)
Married	113 (56.8%)
Never married	4 (2.0%)
Separated	2 (1.0%)
Widowed	56 (28.1%)
Living Location	
Assisted living/Nursing facility	8 (4.0%)
Independent living facility	40 (20.1%)
Living in own home	150 (75.4%)
Living in someone else’s home (friend, relatives, etc.)	1 (0.5%)
Using a cane for home or community ambulation	18 (9%)
Prior Faller (self-reported ≥1 fall(s) in the previous 6 months)	44 (22.1%)
Saint Louis University Mental Status Examination (SLUMS)	26 [23, 27]

Data presented as mean (SD), median [Q1, Q3], or N (%).

**Table 2:** Performance variables (N = 199).

Assessment	Summary Statistic
Berg Balance Scale (BBS), points	54 [51, 55]
Timed Up and Go (TUG), single-task, seconds *	10.3 (9.9, 10.8)
Timed Up and Go (TUG), dual-task, seconds *	11.7 (11.2, 12.2)
Ten Meter Walk Test (10MWT), comfortable, m/s	1.14 (0.24)
Ten Meter Walk Test (10MWT), fast, m/s	1.48 (0.35)
Functional Ambulation and Stair Test, single-task, seconds *	15.2 (14.7, 15.8)
Functional Ambulation and Stair Test, dual-task, seconds *	16.5 (15.9, 17.2)

Data presented as mean (SD), median [Q1, Q3], or for outcomes quantified with an LMM (*), median (95% CI).

## Data Availability

The data that support the findings of this study are available from the corresponding author upon reasonable request.

## Abbreviations

The following abbreviations are used in this manuscript:

FAST	The Functional Ambulation and Stair Test
BBS	Berg Balance Scale
TUG	Timed Up and Go
10MWT	Ten Meter Walk Test
DTC	Dual-Task Cost
SLUMS	Saint Louis University Mental Status Examination
LMM	Linear Mixed Models
SD	Standard Deviation
BMI	Body Mass Index
CI	Confidence Interval
ST	Single task
DT	Dual task
